# Effects of self‐efficacy, self‐esteem, and disability acceptance on the social participation of people with physical disabilities: Focusing on COVID‐19 pandemic

**DOI:** 10.1002/brb3.2824

**Published:** 2022-11-30

**Authors:** Hyeong‐min Kim

**Affiliations:** ^1^ Department of Occupational Therapy Gimcheon University Gimcheon South Korea

**Keywords:** COVID 19 pandemic, degree of disability acceptance, people with physical disabilities, self‐efficacy, self‐esteem, social participation

## Abstract

**Purpose:**

The objective of this study was to understand the effects of self‐efficacy, self‐esteem, and the degree of disability acceptance of people with physical disabilities living in COVID‐19 pandemic on their social participation.

**Methods:**

Among the 4577 registered disabled people who participated in the 2nd wave of the Panel Survey of Employment for the Disabled (PSED), 1682 people with physical disabilities who faithfully answered all the survey items were selected as the final study subjects. This study used the variables of social participation, self‐efficacy, self‐esteem, and disability acceptance, which were validated by experts’ review and consulting and research at the Korea Employment Agency for Persons with Disability. Pearson's correlation analysis and multiple linear regression analysis were performed to identify variables that could predict the social participation of the study subjects.

**Results:**

The self‐efficacy, self‐esteem, and the degree of disability acceptance of people with physical disabilities were positively correlated with social participation. The results of this study showed that self‐efficacy, self‐esteem, the degree of disability acceptance, economic activity, and education level explained 22.4% of social participation.

**Conclusions:**

It was found that self‐efficacy, self‐esteem, and the degree of disability acceptance of people with physical disabilities were important variables affecting social participation. These psychoemotional variables shall be considered for intervention approaches to improve the social participation of people with physical disabilities in the future.

## 1 INTRODUCTION

The United Nations (UN) indicated that it would be necessary to provide consideration in wide sectors, such as employment, protection from violence, and humanitarian support, in addition to health, community facility environment, support services, and education sectors, for people with disabilities during the COVID‐19 crisis (UN, 2020). However, in terms of health rights, people with disabilities face the reality of more severe health inequality than people without disabilities during the crisis when there are risks of isolation, COVID‐19 infection, and side effects and mortality induced by COVID‐19 (Courtenay & Perera, 2020). As a result of a survey by the National Human Rights Commission of the Disabled, 27.2% of 1000 people disabled in this form answered that they experienced discrimination in community participation due to the COVID‐19 pandemic (Lee & Kim, 2020). According to the PSED conducted by the Republic of Korea, 61.5% of the respondents said that they were unable to participate in social activities, indicating that many people with disabilities are restricted from participating in society (KEAD, 2019).

The UN (2020) proposed non‐discrimination, intersectionality, participation, accessibility, accountability, and data disaggregation as the principles of response and recovery for people with disabilities during COVID‐19 (UN, 2020). This is an approach based on the human rights model of the Convention on the Rights of Persons with Disabilities. It emphasizes the need for social participation for citizens with disabilities in all plans and social efforts to respond to and recover from COVID‐19. The International Classification of Functioning, Disability and Health (ICF), proposed by the World Health Organization (WHO), presents social participation and activities as a comprehensive health concept along with physical functions and structures (American Occupational Therapy Association, 2020; WHO, 2001). The AOTA also emphasized the importance of social participation through the Occupational Therapy Practice Framework‐4 (OTPF‐4) (AOTA, 2020; WHO, 2001). As shown, social participation is recognized as a key concept for health in rehabilitation, and its importance is emphasized even more recently (Baum, 2003; Chang et al., 2013).

Social participation is also the key right for people with physical disabilities that must be guaranteed for them to lead an appropriate humane life (Kim et al., 2016). It is very meaningful to the disabled to the extent that it is considered a criterion for producing the pivotal result of successful rehabilitation (Levasseur et al., 2004). However, physical disabilities that experience impairment of physical function make it difficult to recognize the disability or to participate in employment, economic activities, leisure activities, and self‐help activities due to extreme psychological difficulties after an accident (Heo & Park, 2016). As people with physical disabilities repeatedly experience personal or social defeat, they fail to develop adequate self‐esteem and self‐efficacy (Moozova et al., 2015). At the same time, they may show passive and maladaptive behaviors or reveal social dependence (Moozova et al., 2015). They may also have lower self‐esteem than the general population due to awareness of their qualitatively undervalued social identity and restrictions on normal social participation (Baumeister & Leary, 1995). Particularly, people with acquired physical disabilities experience more severe stress to accept their disabilities and overcome them and suffer from more psychological and emotional difficulties than people with congenital disabilities (Priestley, 2003).

To review previous studies related to the social participation of people with physical disabilities, they investigated quality of life (Kim et al., 2016; Levasseur et al., 2004), school segregation (Finnvold, 2018), social support and happiness (Kim et al., 2021), employment (Shin, 2008), and family function (Shin & Kwak, 2008). While studies on the positive function of social participation and ways to increase the level of social participation have been lively conducted (Oh, 2022), studies that consider the psychoemotional aspects of people with disabilities are insufficient. Recent studies that considered the psychoemotional aspects include the study on employment status of people with developmental disabilities (Kim, 2020), the study on the quality of life of people with developmental disabilities (Lee & Lee, 2022), the study on job satisfaction among middle‐aged and older workers with disabilities (Kim, 2019), and the study on emotional awareness level and stress among stroke‐disabled persons (Lim & Yoo, 2019); however, studies on the psychoemotional aspects of people with physical disabilities are insufficient.

Johnston reported that trying an intervention approach considering a negative emotional aspect increases the level of perceptions in the process of recovery, and has a positive effect on the restriction of activity and participation (Johnston et al., 2007). As such, the emotional level is one of the very important factors to improve the level of social participation, and since it can be the basis of social disability, much attention is required (Scott et al., 2012). Recently, studies on the positive function of social participation and ways to increase the level of social participation have been actively conducted (Oh, 2022), but studies considering the psychoemotional aspects of people with disabilities who experience restrictions on social participation are quite insufficient.

Therefore, this study focused on investigating the psychoemotional factors affecting the social participation of the physically disabled. In particular, it aimed to examine how the self‐efficacy, self‐esteem, and degree of disability acceptance of people with physical disabilities affect their participation in society. Through this, it will provide basic data necessary for health promotion of people with disabilities who are passively living in the era of COVID‐19.

## 2 METHODS

### 2.1 Subjects and survey methods

The subjects of this study were people with physical disabilities who participated in the fifth survey of the second wave of the PSED conducted by the Korea Employment Agency for the Disabled. The survey was conducted on the economically active population between 15 and 64 years living in South Korea among registered people with disabilities according to the Act on Welfare of Persons with Disabilities. There were 4577 registered people with disabilities who had one of the 15 disability types prescribed in the Act on Welfare of Persons with Disabilities. This study analyzed 1682 people with physical disabilities who faithfully responded to all items that met the design of this study.

This study primarily conducted tablet PC‐assisted personal interviewing (TAPI) using a tablet PC. It conducted the TAPI program according to the logic design which was previously created and could minimize errors or mistakes that could be made by the investigator by setting the automatic divergence for questionnaire items with complex structures by allowing the investigator to immediately detect wrong responses. The ivestigator initially consulted with the panels on the visiting schedule in advance, and then, /he/she visited them and conducted face‐to‐face investigation with them and entered the responses the tablet PC in the same sentence for clarity (KEAD, 2022).

In the event that face‐to‐face interviews were refused for the reason of COVID‐19 pandemic, online and telephone surveys were conducted. In the case of the telephone survey, a surveyor directly inputted responses into a tablet PC, and if a participant had difficulty in continuing the survey due to a prolonged call, the survey was stopped and was resumed later based on the respondent's schedule. In the case of the online survey, it was carried out with the same questions as the face‐to‐face interview. The questions comprised a total of 11 domains including basic panel information, efforts and support for employment, employment attitude and environment, and daily life and quality of life. The on‐line survey was conducted by providing an individual URL for panels to participate in it, and in the process, the investigator waited to immediately respond to their questions.

### 2.2 Analysis data

The PSED is a national statistic for understanding the status and characteristics of the economic activities of South Koreans with disabilities. It produces dynamic basic statistics related to the economic activities of the disabled. It provides in‐depth and multidimensional information that cannot be generated by conventional cross‐sectional studies by following up on the same individual or group. The plan to conduct the second wave of the PSED, applied in this study, was established in 2015. The first survey of the second wave, the second survey of the second wave, the third survey of the second wave, the fourth survey of the second wave, and the fifth survey of the second wave were implemented in 2016, 2017, 2018, 2019, and 2020, respectively. This study used the raw data from the fifth survey of the second wave in 2020, which reflected social changes and employment issues related to COVID‐19 (KEAD, 2022).

The survey comprises a total of 11 domains including basic panel information, economic activity status determination, wage workers/non‐wage workers/unemployed, efforts and support for employment, occupational ability, employment attitude and environment, daily life and quality of life, and general household matters.

### 2.3 Social participation

The dependent variables applied in this study were variables related to social participation in daily life and quality of life items among the items of the PSED. Social participation refers to participation in formal or informal social groups or gatherings and/or ceremonies such as religious events, graduation ceremonies, and weddings. It was measured on a 4‐point Likert scale by using a response to a single question (“To what extent do you feel you are participating in these ceremonies and activities?”). A higher score indicates a higher degree of social participation. The Cronbach's alpha value of this study was .749.

### 2.4 Self‐efficacy

This study used the 10 items of general self‐efficacy, which were developed by Schwarzer and Jerusalem (1995) and translated into Korean and validated by Lee (1994). The response categories consist of a total of 4 points in the Likert scale with 1: not at all, 2: disagree, 3: yes, and 4: strongly agree, with higher scores indicating higher self‐efficacy. The Cronbach's alpha of this study, indicating the reliability, was .909.

### 2.5 Self‐esteem

This study used the 10 self‐esteem items that were developed by Rosenberg (1965) and translated by Jun (1974). The response categories are 1 (not at all), 2 (disagree), 3 (yes), and 4 (strongly agree) on a 4‐point Likert scale. A higher total score means a tendency of considering one's own values more positively. The Cronbach's alpha value of this study was .682.

### 2.6 Degree of disability acceptance

For the degree of disability acceptance, this study used 12 items: nine Disability Acceptance Scale (DAS) items (Kaiser et al., 1987), and three items for factors to overcome disabilities of the self‐acceptance test, which was developed by Kang et al (2008). The response categories are 1 (not at all), 2 (disagree), 3 (slightly agree), 4 (somewhat), and 5 (strongly agree) on a 5‐point Likert scale. A higher score indicates that people with disabilities have a stronger tendency to accept life with disabilities with their own subjectivity. The reliability of the disability acceptance scale items was measured by Cronbach's alpha .776.

### 2.7 Statistical analysis

The data of this study were analyzed by using SPSS WIN 21.0. The general characteristics of the study subjects were analyzed by descriptive statistics. Pearson's correlation was used to analyze the correlation between general characteristics of people with physical disabilities, self‐esteem, self‐efficacy, the degree of disability acceptance, and social participation. Finally, a multiple linear regression analysis was performed to examine the effect of each variable on social participation. Statistical significance was determined at alpha = .05.

## 3 RESULTS

### 3.1 General characteristics of subjects

This study confirmed the sociodemographic characteristics of the respondents. As a result of analysis, it was found that as for the sex of people with physical disabilities, males were 1147 (68.2%) and females were 535 (31.8%). As for age, analysis showed that 81 persons (4.8%) belonged to the age of 15–29, 221 persons (13.1%) to the age of 30–39, 557 persons (33.1%) to the age of 40–49, 478 persons (28.4%) to the age of 50–59, and 345 persons (20.5%) to the age of 60–67, respectively. As for the level of education, it was found that middle school graduates or below were 398 (23.7%), high school graduates 796 (47.3%), and college graduates 488 (9.0%), respectively, and for severity of disability, those with mild disability were found to be 1467 (87.2%) and those with severe disability were found to be 215 (12.8%). As for economic activity, the employed were found to be 1072 (63.7%) and the unemployed were found to be 610 (36.3%) (Table 1).

**TABLE 1:** General characteristics of study subjects (*N* = 1682)

Characteristics	Classification	Frequency (persons)	Percentage (%)
Gender	Male	1147	68.19
	Female	535	31.81
Age	15–29	81	4.82
	30–39	221	13.14
	40–49	557	33.12
	50–59	478	28.42
	60–69	345	20.51
Education level	Middle school and under	398	23.66
	High school	796	47.32
	University and above	488	29.01
Disability severity	Mild	1467	87.22
	Severe	215	12.78
Economic activity	Employed	1072	63.73
	Unemployed	610	36.27

### 3.2 Correlation between variables influencing social participation

This study conducted Pearson's correlation analysis to understand the correlation between the variables affecting social participation. This study included age, education level, economic activity, and the severity of disability, which significantly affected social participation, in correlation analysis among the sociodemographic characteristics of people with physical disabilities. Analysis results showed that educational level (*r* = .243, *p* < .001), disability severity (*r* = .117, *p* < 001), self‐efficacy (*r* = .400, *p* < .001), self‐esteem (*r* = .350, *p* < .001), and the degree of disability acceptance (*r* = .336, *p* < .001) were positively correlated with social participation. Moreover, it was negatively correlated with age (*r* = ‐.105, *p* < .001) and economic activity (*r* = ‐.323, *p* < .001) (Table 2).

**TABLE 2:** Correlation between variables affecting the social participation (*N* = 1682)

	Social participation	Age	Education level	Economic activity	Severity of disability	Self‐efficacy	Self‐esteem
Age	–.105***						
Education level	.243***	–.455***					
Economic activity	–.323***	.146***	–.302***				
Severity of disability	.117***	.045	.031	–.143***			
Self‐efficacy	.400***	–.109***	.275***	–.443***	.186***		
Self‐ esteem	.350***	–.075**	.194***	–.330***	.138***	.498***	
Acceptance of disability	.336***	–.067**	.219***	–.364***	.143***	.531***	.461***

**p < .01, ***p < .001.

### 3.3 Causal relationship of variables influencing social participation

Stepwise multiple linear regression analysis was carried out to identify variables influencing the social participation of people with physical disabilities. Multicollinearity between independent variables was analyzed using tolerance and variance inflation factor (VIF). The results showed that tolerance was .10 or higher and VIF was less than 10. Therefore, it was found that there was no multicollinearity. Durbin–Watson test was carried out to verify the independence of the residuals. The results showed that the *d* value was 1.465, indicating that there was no autocorrelation. This study analyzed the variables affecting the social participation of people with physical disabilities to find that the regression model was suitable (*F* value = 98.147, *p* < .000) and the explanatory power was 22.4% (adj. = 0.224). Self‐efficacy (*β* = .029, *p* < .001), self‐esteem (*β* = .033, *p* < .001), economic activity status (*β* = −.183, *p* < .001), education level (*β* = .103, *p* < .001), and the degree of disability acceptance (*β* = .013, *p* < .001) significantly influenced the social participation of people with physical disabilities (Table 3).

**TABLE 3:** Factors affecting the social participation (*N* = 1682)

	Unstandardized coefficients		Standardized coefficients	*t*	Tolerance	VIF
	B	Std. error	Beta			
Constant	.203	.154		2.717**		
Self‐efficacy	.029	.004	.191	6.776***	.582	1.717
Self‐esteem	.033	.006	.150	5.794***	.691	1.448
Economic activity	–.183	.037	–.122	–4.895***	.744	1.345
Education level	.103	.023	.104	4.530***	.881	1.135
Acceptance of disability	.013	.004	.098	3.696***	.652	1.533
*R* ^2^	.224					
*F* (*p*)	98.147 (.000)					

^**^ *p* < .01; ***p < .001.

## 4 DISCUSSION

People with disabilities living in the era of COVID‐19 are experiencing substantial barriers to educational opportunity and social participation, which they should be able to enjoy, in addition to minor prejudices that they encountered in daily life. The role of helping active social participation within the context of each individual person with disabilities can be a major task in the field of rehabilitation. Therefore, it is necessary to take a comprehensive approach, which can improve functional independence, community integration, and quality of life by identifying factors that promote functional recovery of the disabled and social participation activities (Kim & Cho, 2013). Among them, the psychoemotional aspect is recognized as an important factor in the social interaction and participation of the disabled (Gross, 2002). In particular, the psychoemotional variable of physically disabled persons should be importantly considered in the process of rehabilitation, as it can be directly connected with spontaneous behavior of the disabled, and therefore, with their successful social participation. This study, considering these aspects, identified the effects of the self‐efficacy, self‐esteem, and the degree of disability acceptance perceived by people with physical disabilities on their social participation, and it will discuss mainly these results.

The PSED data is useful for understanding the entry and movement in the labor market, the characteristics of economic activities according to the life cycle, and changes in employment patterns and preferences of people with disabilities (KEAD, 2022). It is possible to comprehensively identify all factors related to the establishment and evaluation of employment policies for people with disabilities through this. The data of the 5th survey conducted in 2020 reflected social responses, social changes, and pending employment issues in relation to COVID‐19. This study analyzed relevant variables based on the fifth survey data of the second wave of the PSED to identify the variables affecting the social participation of people with physical disabilities. The correlation analysis confirmed the positive correlation between the dependent variable social participation and the independent variables, respectively, self‐efficacy, self‐esteem, and the degree of disability acceptance. Moreover, this study analyzed the causal effects of the sociodemographic characteristics, self‐efficacy, self‐esteem, and the degree of disability acceptance of people with physical disabilities, which were independent variables, on social participation. The results showed that self‐efficacy, self‐esteem, economic activity, education level, and the degree of disability acceptance explained 22.4% of social participation.

The B value of self‐efficacy was positive (.029), indicating that better self‐efficacy increased the degree of social participation. This result of this study support Yang et al (2017), which confirmed a significant relationship between self‐efficacy and social participation of people with physical disabilities. Self‐efficacy is defined as the belief that an individual believes that he or she has the ability to perform a special task by bringing out one's potential under adverse circumstances (Bandura, 1986). People with disabilities set higher standards and goals, invest more effort, and do not give up easily even in a difficult situation owing to self‐efficacy (Klassen & Usher, 2010). As shown, the issue of whether recognizing oneself as a valuable existence is a key variable for increasing the social participation of people with physical disabilities in the era of COVID‐19. According to a recent study, it was verified that self‐efficacy acts as a mediator variable in the relation between digital literacy and social isolation among people with disabilities (Park et al., 2022). This result suggests the importance of information accessibility for people with disabilities who experience difficulty in information acquisition after the COVID‐19 outbreak, and proves that the management of self‐efficacy through digital literacy can be an important variable capable of explaining social participation. Therefore, it may safely be said that it can be methods for enhancing the self‐efficacy and social participation of people with physical disabilities for them to be exposed to diverse media through the use of digital platforms and apply them to their life.

The B value of self‐esteem was positive (.033), indicating that higher self‐esteem increased the degree of social participation. These results agreed with the results of Ko's (2015), which showed that self‐esteem had a significantly positive impact on social participation. The result revealed that self‐esteem played a positive role in social participation. Kim and Nam (2022) reported that self‐esteem was significantly reduced among the disabled community due to COVID‐19. The reduction of self‐esteem is likely to deepen social isolation, which can cause considerable constraints on social participation. Recent studies on rehabilitation psychology conducted in the United States suggested an approach that emphasizes self‐esteem and identity recovery through social participation (Bogart, 2015; Forber‐Pratt & Zape, 2017). In the context of the COVID‐19 pandemic, the preparation of methods for enhancing the self‐esteem of people with physical disabilities and the society's concern for them will be helpful in their voluntary social participation.

In the midst of the COVID–19 crisis, people with disabilities not only experience health and social inequality, but also face stigma and prejudice due to disability, feeling helpless that there is nothing they can do for themselves, and being unable to respond to the spread of the COVID–19 virus. (Inter Agency Standing Committee, 2020). Social stigma and discrimination based on disability make people with disabilities experience frustration and make it difficult for them to accept their disabilities (Bogart, 2015). This study analyzed the relation between disability acceptance and social participation in consideration of this; and the results of analysis showed that the higher the degree of disability acceptance (*B* = .013), the higher the degree of their social participation. It supported the results of previous studies (Oh, 2022; Park & Yang, 2015), which revealed that the social participation of people with acquired physical disabilities significantly affected disability acceptance. Previous studies on the process of accepting disability (Dunn & Burcaw, 2013; Forber‐Pratt & Zape, 2017) consistently reported that people with the highest acceptance level actively participated in social activities. The receptive attitude toward disabilities promotes active participation in society (Hahn & Belt, 2013). The experience of accepting own disability, discovering own value in relationships with others with disabilities, and active social participation is the foundation for forming a positive self‐concept (Forber‐Pratt & Zape, 2017). As a result, disability acceptance, which is greatly affected by subjective aspects such as the cognitive and psychological factors of people with physical disabilities, should be emphasized academically (Lee & An, 2011).

COVID‐19 has a great impact on the micro‐sphere of every individual as well as on the macro aspects of national economy. In particular, constraints on social participation and psychoemotional problems experienced by people with disabilities can be remarkable factors in the area of rehabilitation. Since the beginning of the COVID‐19 pandemic, the number of studies dealing with the psychological consequences is growing rapidly (Mergel & Schützwohl, 2021). This study also reflects this trend of the times, and psychoemotional factors such as self‐efficacy, self‐esteem, and disability acceptance perceived by the people with disabilities amid the COVID‐19 pandemic are important factors that cannot be ignored for their social participation and satisfactory life.

The importance of this study was to seek the intervention strategies of rehabilitation for enhancing the social participation of people with physical disabilities by examining factors influencing their social participation, as social interest in the healthy life of people with physical disabilities has increased after the COVID‐19 pandemic. Despite the importance of this study, this study had several limitations. First, since this study analyzed secondary data, there was a limit in measuring each variable and generalizing the value. Future studies need to evaluate the relationship with participation in social activities by collecting data using a standardized scale that reflects the specificity of diseases. Second, in the case of the variable of social participation, it is difficult to verify reliability with a single question item. In this study, panel survey data based on longitudinal analysis were used, and the panel retention rate of the PSED is 86.3%. Given this, reliability was presented by verifying it with time difference between the responses of panel survey data. That is, reliability was verified by the cross‐validation of the data of the third, the fourth, and the fifth waves of PSED. In future studies, their research design will have to be carried out in consideration of the limitations of this study.

## 5 CONCLUSIONS

This study aimed to confirm factors influencing the social participation of people with physical disabilities living in the era of COVID‐19 based on the PSED data. It was found that self‐efficacy, self‐esteem, and the degree of disability acceptance were variables affecting the social participation of people with physical disabilities. Efforts to improve the self‐esteem, self‐efficacy, and disability acceptance are essential to increase the social participation of people with physical disabilities. Based on such factual basis, it is necessary to consider psychoemotional factors when approaching interventions to enhance social participation of people with disabilities living in the community

## AUTHOR CONTRIBUTION

The author was involved in the conception and design of this study, the analysis and interpretation of the data, and drafted and revised the article.

## CONFLICT OF INTEREST

The author declared no potential conflicts of interest with respect to the research, authorship, and publication of this article.

### PEER REVIEW

The peer review history for this article is available at https://publons.com/publon/10.1002/brb3.2824

## Data Availability

The data used to support the findings of this study are available from the corresponding author upon request.
